# Translation and cross-cultural adaption of the Chinese version of the Vanderbilt Head and Neck Symptom Survey version 2.0: a tool for oral symptom assessment in head and neck cancer patients

**DOI:** 10.1186/s12955-021-01673-4

**Published:** 2021-01-21

**Authors:** Min Jin, Li Sun, Rui Meng, Wenjing Wang, Rui Sun, Jing Huang, You Qin, Bian Wu, Qian Ding, Gang Peng, Tao Zhang, Kunyu Yang

**Affiliations:** Cancer Center, Union Hospital, Tongji Medical College, Huazhong University of Science and Technology, Wuhan, 430022 China

**Keywords:** Translation, Oral symptoms, Quality of life, Head and neck cancers, VHNSS 2.0

## Abstract

**Background:**

Patients with head and neck cancer (HNC) who are receiving radiotherapy commonly face detrimental complications, including oral issues. However, oral symptoms are not well understood given the lack of available specific assessment instruments. The Vanderbilt Head and Neck Symptom Survey version (VHNSS) 2.0 is an instrument specifically developed to identify oral symptoms in HNC patients receiving radiotherapy in the United States.

**Objective:**

To perform the translation and cross-cultural adaptation of the original English version of VHNSS 2.0 into a Chinese version (Mainland China).

**Methods:**

The translation and cultural adaptation process involved translation by independent translators, construction of a consensus version, back translation into the original English version, analysis by the expert committee and a pretest. The pretest was administered to 90 patients with HNC to assess the feasibility and practicality of the tool.

**Results:**

The final Chinese version approved by the expert committee was well understood by all participants in the study. The instrument had satisfactory content validity, with indexes of 0.83 for semantic and idiomatic equivalence, 0.90 for cultural equivalence, and 0.91 for conceptual equivalence. Furthermore, this version had good internal consistency, with Cronbach's alpha coefficients ranging from 0.74 to 0.95.

**Conclusion:**

The Chinese version of VHNSS 2.0 was translated and cross-culturally adapted for use in China. This translation is a feasible instrument to assess oral health-related quality of life in HNC patients undergoing radiotherapy and will be useful for symptom management by clinicians and researchers in China.

## Introduction

Head and neck cancer (HNC), a heterogeneous group of upper aerodigestive tract malignancies, is the sixth most common cancer worldwide [1]. Each year, an estimated 560,000 new cases of HNC (including cancer of the oral cavity and pharynx, tongue, mouth, pharynx and other oral cavity regions) are diagnosed and an estimated 300,000 deaths [2]. The standard treatment for patients with HNC is surgery, radiotherapy (RT), chemotherapy, targeted therapy or a combination of these treatments [3, 4]. With the implementation of treatment strategies for the anatomical preservation of the structures, RT has been the most important therapeutic modality that is used to treat approximately 80% of the patients with HNC [5]. Despite enormous advances in RT planning and delivery, the disease and its treatment still have a disproportionate impact on a patient’s quality of life (QOL) [6, 7]. For example, treatment of HNC with radiotherapy often results in highly visible disfigurement and disruptions of essential functioning, such as deficits or complications in eating, swallowing, sleep, and speech. In particular, oral alterations are notable and include xerostomia, mucositis, taste and smell sensitivity alterations, dysphagia, excess mucus, reduced mouth opening and tooth alterations [8–10]. A large proportion of patients with HNC survive after treatment, but some of the treatment sequelae influence the survivors’ QOL for a long time. HNC is believed to be the most psychologically traumatic cancer to experience. Thus, clinical administrators collaborate in a multidisciplinary team to develop cancer management plans and address treatment related side effects to improve survivors' QOL.

Although many instruments for the evaluation of QOL and psychosocial functioning exist, most are unavailable in the Chinese language or only include a few items for oral symptom assessment in HNC patients. The University of Washington Quality of Life Questionnaire (UW-QOL) [11, 12], the Functional Assessment of Cancer Therapy Head and Neck Scale (FACT-HN) [13] and the European Organization for Research and Treatment of Cancer Quality of Life Questionnaire-H&N35 (EORTC QLQ-H&N35) are the most frequently used tools to assess QOL in HNC [14]. None of these instruments are specifically designed to screen for oral-related problems and are inconvenient for clinical practice. To date, no standard instrument has been developed for the assessment of oral-related symptoms. In 2009, the Vanderbilt Head and Neck Symptom Survey (VHNSS) was designed by Murphy et al. to provide an expanded assessment of oral health symptoms in the HNC population of the United States [15]. The survey includes 28 items and excludes questions that address general symptom control issues, such as fatigue, nausea/vomiting, and gastrointestinal symptoms, because they are not specific to HNC. However, some potential important adverse effects were insufficient or not included in VHNSS, such as mucosal sensitivity, dental health and truisms. Thus, VHNSS 2.0 was further developed from the original version [16]. The revised VHNSS 2.0 includes 50 items within 13 domains. The items are scored on a numeric scale rating the severity of the symptom from 0 (none) to 10 (severe). The VHNSS demonstrated adequate psychometric properties, including validity, reliability and sensitivity, and is a valid and reliable tool to identify HNC-specific symptom burden.

In 2014, VHNSS 2.0 was translated and cross-culturally adapted into Brazilian Portuguese [17], and the Brazilian Portuguese translated version presented good results for convergent validation and known-group analysis [18]. Most recently, VHNSS 2.0 was also translated and cross-culturally adapted into an Italian version [19], further demonstrating excellent feasibility and utility [20]. Thus, the VHNSS 2.0 scale is of great value to identify HNC-related symptoms in routine clinical practice. To date, the VHNSS 2.0 has never been validated and applied in Asian countries, making its universality restricted.

The goal of this study was to translate and cross-culturally adapt the original United States version to Chinese (Mainland China) according to a standardized, well-established procedure [21, 22] and conduct a pretest of the survey in HNC patients receiving radiotherapy to assess its simplicity and clarity.

## Methods

### Design and procedure

**Fig. 1:**
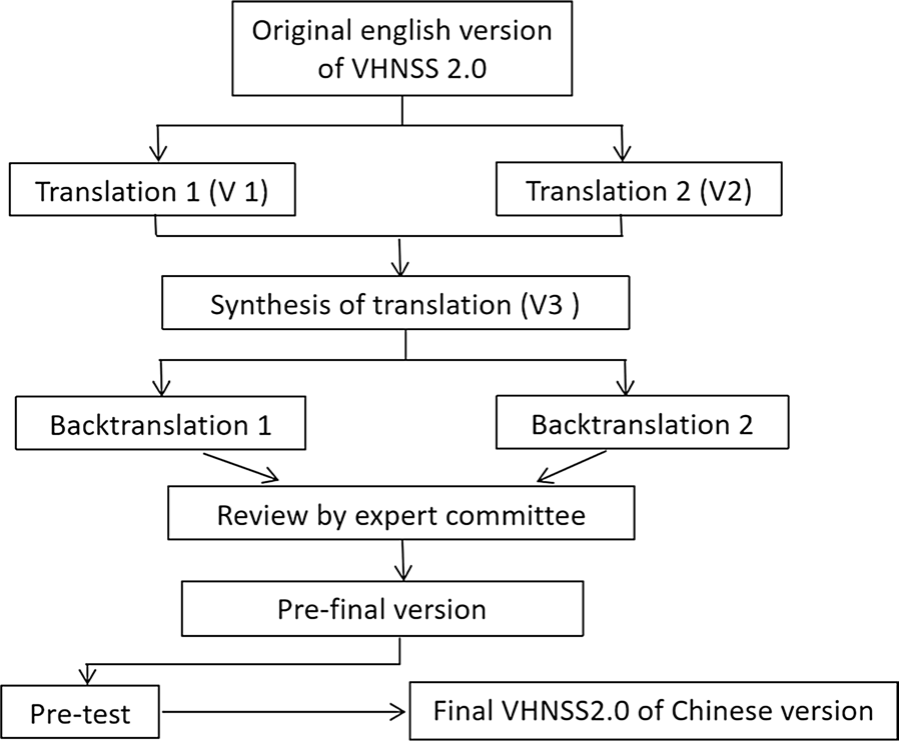
Flowchart of the process of translation and cross-cultural adaptation of of the Vanderbilt Head and Neck Symptom Survey version 2.0 for use in China

This study was conducted in two phases. In the first phase, forward-translation, back-translation, and cultural adaptation were conducted to finalize the draft Chinese version of VHNSS 2.0. In the second phase, the oral health outcomes of VHNSS 2.0 were pretested using a cross-sectional survey. Consent and permission to use the VHNSS 2.0 of the scale were obtained by email from the original author Dr. Barbara A. Murphy. The study was approved by the Institutional Review Board of Huazhong University of Science and Technology. The steps of the translation and cross-cultural adaptation process are shown in Fig. 1.

### Phase I: forward translation, back translation, and cultural adaptation

#### Step 1: forward translation

Two native Chinese translators who are proficient in English independently translated the original version of VHNSS 2.0 into Chinese versions (V1 and V2) according to the standard process for translating instruments. One of the translators has a doctorate in oncology and is familiar with HNC-related terms. The other translator is a postgraduate student who majored in nursing. After obtaining the two Chinese versions, a professional translator and the first author checked the translations and synthesized them into one document. Discussions were held among the translators to achieve consensus when controversy regarding statements or ambiguity related to wording occurred.

#### Step 2: back translation

The Chinese version was translated back into English by two separate bilingual professors who had not previously viewed the scale. The back-translated version was coordinated and discussed by the professional translators to ensure equivalence and consistency. Changes to sentences and words were approved to obtain the back-translated version.

#### Step 3: cultural adaptation (an expert panel and pilot testing)

An expert committee comprising a panel of five experts (including two oncologists, a nursing professor, and two senior nurses engaged in HNC) reviewed the items of the Chinese version and compared it to the original version. For the content validity index (CVI), experts needed to rate each item of the instrument concerning semantic/idiomatic, cultural and conceptual aspects based on scoring (− 1, 0 or 1). Score “0” (zero) represented a somewhat equivalent item with some vague expressions, “− 1” (minus one) represented a nonequivalent item, and “1” (one) represented a highly equivalent item. The item-level CVI (sum of scoring divided by the number of total experts involved) was calculated. Based on this value, the scale-level CVI for the overall scale (sum of all items CVI divided by the total number of items) was accordingly determined [17, 23]. The content was regarded as equivalent when CVI > 0.8 [23]. After the data were analyzed, it was determined whether revisions were necessary to accommodate the experts’ feedback. If major revisions were needed, the process was repeated. Finally, a new prefinal version was obtained.

### Phase II: pretest of the Chinese version of the VHNSS 2.0

We conducted a pretest on 90 participants to assess the clarity and understandability of the final version as well as internal consistency of the items via the Cronbach's alpha coefficient. The formula used to calculate the sample size for the Cronbach alpha test was as follows [24]:

$$n = \left[ {{{\left\{ {\left( {\frac{2k}{{k - 1}}} \right)\left( {Z_{{{\raise0.7ex\hbox{$\alpha $} \!\mathord{\left/ {\vphantom {\alpha 2}}\right.\kern-\nulldelimiterspace} \!\lower0.7ex\hbox{$2$}}}} + Z\beta } \right)^{2} } \right\}} \mathord{\left/ {\vphantom {{\left\{ {\left( {\frac{2k}{{k - 1}}} \right)\left( {Z_{{{\raise0.7ex\hbox{$\alpha $} \!\mathord{\left/ {\vphantom {\alpha 2}}\right.\kern-\nulldelimiterspace} \!\lower0.7ex\hbox{$2$}}}} + Z\beta } \right)^{2} } \right\}} {\ln \left( {\frac{{1{\text{ - CA0}}}}{1 - CA1}} \right)^{2} }}} \right. \kern-\nulldelimiterspace} {\ln \left( {\frac{{1{\text{ - CA0}}}}{1 - CA1}} \right)^{2} }}} \right] + 2$$

where n = sample size, k = the number of items, CA0 = the value of Cronbach’s alpha at the null hypothesis (0.5), and CA1 = the expected value of Cronbach’s alpha (0.7). In our study, we set K at 50, CA0 at 0.5, CA1 at 0.7, power at 90% (Power = 1 − β) and the value of α at 0.05. Based on an estimate, the requirement for sample size was 85.

Patients in this study were diagnosed with HNC (nasopharynx, nasal, paranasal sinus, oral cavity, pharynx, or larynx) and had completed radiotherapy for at least one month. We excluded patients who did not express willingness to participate in Chinese and who had cognitive or mental impairment. The recruited subjects agreed to participate by signing informed consent forms. The patients were given the option to either self-administer the tool or have it applied by the interviewer. Given that some patients were illiterate, poorly educated, or do not like reading, the interviewer will read out the questions for them to answer. Thus, the paper-based tool was initially designed for both self-administration and administration by an interviewer. In the process, if the patients had any questions, the interviewer would provide an explanation. All participants were hospitalized, and they completed a questionnaire at one time point in the clinic. Their completion time was also calculated. After completing the questionnaire, participants were asked for a face-to-face interview concerning the clarity and understanding of the scale. The feedback was documented and used for further revision by experts. The draft Chinese version of VHNSS 2.0 was finalized after the above steps.

### Ethical considerations and procedures

The institutional review board of the hospital approved this study. The research procedures and recruiting criteria were explained to researchers before they contacted the potential subjects. All the participants were referred by doctors and provided informed consent for participation in this study. It was emphasized that participation was voluntary and could be withdrawn at any time. The subjects’ responses were considered anonymous and confidential. The researchers explained the risks and benefits of participation as well as the patients’ right to refuse to participate without jeopardizing treatment.

### Statistical analysis

The baseline characteristics of the participants included both categorical and qualitative data. Categorical data, including gender, primary tumor site, TNM stage, treatment and ECOG, were presented as the number (%), and quantitative data, including age, were presented as the mean ± SD. These descriptive statistics were analyzed using SPSS version 22.0 (IBM Corp. 2011, NY, USA). The value of CVI for evaluating the content validity of the questionnaire was calculated using Excel software (Microsoft). Cronbach’s alpha coefficient was used to assess internal consistency by SPSS. A value of CVI > 0.8 was regarded as equivalent to the translated version and original version. A value of Cronbach’s alpha > 0.7 was regarded as indicating good internal consistency.

## Results

### Translation and cultural adaptation

The process of translation and cross-cultural adaptation of the VHNSS 2.0 tool into the Chinese language is described in Fig. 1. In the step of forward-translation, the two forward translators independently translated the original version of VHNSS 2.0 into Chinese (V1 and V2 forward translation). The forward translators declared that the original items were easy to understand and translate. After obtaining the V1 and V2 forward translation, a professional translator and the first author checked the translations and synthesized them into document (V3).

In the back-translation step, the translated version (V3) was translated back into English by two separate bilingual professors who had not seen the original English version of the scale. Next, a discussion was performed by the bilingual translators to ensure equivalence and consistency. After discussion, the experts provided some suggestions that were incorporated into the tool of the translated version based on the Chinese culture habit. Table 1 shows the main cultural adjustments within the items and tool responses. Notably, all the items in the translated version are in the 2nd person singular, whereas the original version is in the 1st person singular. A major consideration is that China is a country with a multiethnic population and varied education levels. In our cancer center, some HNC patients were old and poorly educated or did not like reading, so they would prefer the investigator to read the questions to them to answer in the 2nd person. Additionally, the modification using the 2nd person singular provides more clarity and is appropriate for both self-application and application by the investigator.

**Table 1:** Modifications suggested for VHNSS 2.0 items by the expert committee and translators

Item	Original version	Back-translation	Translated and adapted version (Chinese)	Rationale
1^st^ Directions	By checking the appropriate box	by Choosing an appropriate option	通过选择一个合适的选项	Clearer and more informal
1	I currently have a feeding tube in place	Are you using a feeding tube in place now	你正在使用营养饲管吗	Clearer and more informal
2^nd^ Instruction	In general, a “0” indicates the least amount of problems with a particular symptom and “10” indicates the most problems	In general “0” indicates the absence of a symptom and “10” indicates the maximum presence of symptom	一般来说, 0表示没有相关症状, 10表示症状最严重	The substitution of the term “amount” by “presence” makes the sentence most objective
3	like Ensure® or Boost®	Like milk or nutrison	比如牛奶或能全素	In China, Ensure® or Boost® are rarely seen in the market. we commonly use milk or nutrison for nutritional supplement
6	like water, tea and Ensure®	like Plain boiled water, tea and milk	比如白开水, 茶或者牛奶	More informal word, culturally appropriate
16	Problems with dry mouth affect my ability to sleep	Problems with dry mouth make you unable to sleep well	口干导致你睡眠不好	Facilitates understanding
27,39, 40, 41, 42, 43	Not applicable	Not applicable for you	不适用你	Clearer
49	I have limitations in the ability to open or move my jaw	You have limitations in the ability to open your mouth or move your jaw	你张口或者移动下颌会受限	Facilitates understanding and Culturally adequate

VHNSS 2.0, Vanderbilt Head and Neck Cancer Symptom Survey version 2.0

Regarding the CVI, all expert committee members independently scored the fifty items in to obtain the item-level CVI. Three categories were assessed for each item: semantic/idiomatic, conceptual and cultural. The scale-level CVI was calculated based on the item level-scale. Our calculation result showed that the scale-level CVI was 0.83 for idiomatic and semantic equivalence and > 0.9 for the other equivalences (Table 2) with a minimum value of 0.80 for the evaluated item [23].

**Table 2:** Mean of the CVI of the items

Equivalence	CVI of items	CVI of answers to items
Semantic/idiomatic	0.83	0.94
Cultural	0.90	0.95
Conceptual	0.91	0.92

CVI, content validity index

### Pretest

For the pretest, 90 patients diagnosed with HNC after radiotherapy were included. Patients with cognitive or mental impairment were not included in the survey. The shortest time to complete the scale was one month after radiotherapy, and the longest was 5 years after radiotherapy. The median age of the patients was 49.50 years (range, 26–76 years). Among the participants, 64 (71.11%) were male, and 26 (28.89%) were female. Table 3 demonstrates the demographic and clinical characteristics of the study population. The tumor types were nasopharyngeal carcinoma in 69 patients (76.67%), malignancy of nasal and paranasal sinus in 3 (3.33%), oral cancer in 6 (6.67%), hypopharyngeal malignancy in 5 (5.55%) and laryngeal carcinoma in 7 (7.78%). The most frequent pathologic histology type in the present study was squamous cell carcinoma. Most subjects had a good performance status (ECOG PS = 0). The type of treatment and TNM staging (according to the American Joint Committee on Cancer [AJCC]) are shown in Table 2. The average time for patients to complete the scale of the Chinese version of VHNSS 2.0 was 7.5 min with a minimum of 5 min and a maximum of 11 min. In the pretest, 81 patients completed all 50 items, one patient missed 3 items, three patients missed 2 items, and five patients missed one item during their responses. The missing responses pertained to items 26, 17, 31, 38 and 40. These missing items mostly occurred in populations testing by self-administration.

**Table 3:** Patients and tumor characteristics

Characteristic	Value
Age, year	
Median	49.50
25^th^–75th interquartile range	42–55
Range (min, max)	(26, 76)
Gender, no.(%)	
Female	26 (28.89%)
Male	64 (71.11%)
Primary tumor site	
Nasopharynx	69 (76.67%)
Nasal, paranasal sinus	3 (3.33%)
Oral cavity	6 (6.67%)
Hypopharynx	5 (5.55%)
Larynx	7 (7.78%)
TNM stage	
I	2 (2.22%)
II	13 (14.44%)
III	25 (27.78%)
IV	49 (54.45%)
“Missing”	1 (1.11%)
Treatment	
Combined chemoradiation	74 (82.22%)
Radiation only	5 (5.56%)
Combined chemoradiation + surgery	8 (8.89%)
Radiation + surgery	3 (3.33%)
ECOG	
0	63 (71.170.00%)
1	27 (28.930.00%)

TNM, classification of malignant tumors; ECOG, Eastern Cooperative Oncology Group

After a face-to-face interview for the patients’ feedback, all the patients demonstrated that they could easily understand each question, and the answer choices were clear. Among these 90 subjects, 74 did not recommend any change, whereas 16 subjects suggested some minor revisions. Three subjects deleted the first 3 items in the directions because they considered that these items were unnecessary and were repetitive with the following questions to some extent. Three patients were followed-up for five years, and they reported that most of the symptoms had disappeared and suggested simplifying the scale. These suggestions were refused because the proposed changes were inconsistent with the original source. Six patients asked to add a check box separately under each level choice for items, and one patient asked to add the smiley face of pain rating for items 25 and 16. These items were refused because it would affect the esthetics and simplicity of the scale. Five patients asked to change item 38, “You have altered what you eat due to a change in your sense of smell”, to “You have altered which food you choose to eat due to a change in your sense of smell”. This change was retained because it was clearer for patients.

For the reliability of the translated version based on pretest, Cronbach’s alpha showed a high degree of internal consistency and homogeneity between items for subgroups of nutrition (0.89), swallowing/eating foods (0.95), xerostomia (0.87), excess mucus (0.92), mucositis (0.94), pain (0.93), speech/communication (0.93), taste change (0.95), smell (0.85), mucosal sensitivity (0.86) and range of motion (0.74).

## Discussion

In the present study, we successfully translated the original United States version of VHNSS 2.0 into a Chinese version, adapting it to the cross-culture habit of the target country and validating the tool in a pretest of HNC patients receiving radiotherapy. The multistep method of translation and cross-cultural adaptation used in this study has been consolidated in previous studies [17, 19]. To the best of our knowledge, it is the first translated scale in Chinese (mainland) with several domains of multidirectional oral functional assessment in a HNC population following radiotherapy.

During the process of translation and cross-cultural adaptation of the scale, grammatical and cultural adjustments were modified to adapt it to the Chinese context and facilitate the understanding of each item by experts and patients. We changed the first-person singular to the second-person singular, so the instrument could be used through self-assessment or evaluation by interviewers. In our study, 45% of the participants preferred self-assessment of the scale, whereas 55% chose evaluation by interviewers. Social and educational levels as well as age could potentially influence the choice. Some participants had a low educational level with lower income, making it difficult to perform a self-evaluation. Some patients were older and preferred to be assessed by interviews instead of performing a self-evaluation.

Although various tools have been developed to assess complications in HNC patients [25, 26], a focal and available instrument is still lacking for clinicians to evaluate and manage HNC-related problems in China. Thus, the present study was designed and performed. The original VHNSS developed by Professor Barbara A. Murphy aimed to provide a screening tool to help recognize HNC-specific symptoms and control problems in a timely fashion. The revised VHNSS 2.0 containing 50 items distributed into 13 domains is an effective and reliable tool for identifying the specific symptom of HNC [16, 27] and has been translated in Brazilian Portuguese [17] and Italian languages [19]. The original VHSSS exhibited good internal consistency (Cronbach’s alphas 0.74 to 0.95) [16], and this value is similar to that of our translated version. The assessment of equivalence by the expert committee demonstrated that the Chinese version of the VHNSS 2.0 is equivalent to the original United States version.

In the pretest, patients with nasopharyngeal carcinoma (NPC) accounted for most (76.7%) of the participants in our study. NPC is highly prevalent in Southern China with an annual incidence of 30 cases per 100,000 persons [28], which is in contrast to a lower incidence of 1 case per 100,000 persons in Western countries [29]. Additionally, some scholars refer to NPC as a “Chinese cancer”. From the first instruction question “I currently have a feeding tube in place”, we found that few patients used a feeding tube in China. The guidelines strongly recommend enteral feeding by naso-gastric or percutaneous tubes in radiation-induced severe mucositis or with an obstructive HNC mass [30]. Enteral tube feeding is indicated in cases of severe dysphagia and inadequate energy intake [31]. Studies have indicated that hypopharyngeal cancer, particularly T4 tumors, female sex, or combined radiochemotherapy, are high risk factors of dysphagia and more likely to require energy intake by tube feeding [32]. In our study, the percent of patients using feeding tube is relatively low (2.2%) possibly because the number of people with hypopharyngeal cancer constituted only a small minority (5.6%) of the study population or because the identification of those with decreased oral intake who require nutritional intervention is insufficient in the HNC population.

We acknowledge several limitations of this study. The sample size is relatively small and may affect the findings to some extent. Cronbach’s alpha is a test of internal consistency that is typically followed by a factor analysis to establish unidimensionality. Factor analysis was not conducted in the study because this would require a much larger sample size of at least 500 patients. Moreover, the participants were exclusively selected from hospitalized patients in a cancer center, and no other measuring instruments were used. Additional larger studies for administration in clinical practice are needed to further validate the current findings in the future.

## Conclusions

Translation and cultural adaptation of the VHNSS 2.0 tool into Chinese (Mainland China) has been successfully performed in this study, revealing its use as an important tool to assess oral symptoms in HNC patients in China. Our results demonstrated that the Chinese version of the VHNSS 2.0 tool is equivalent to the original tool in English. The translation is easily understood by patients and adapted to Chinese culture. It is hoped that it can offer help for Chinese clinicians and researchers to evaluate oral alterations in a timely manner and develop strategies to manage such changes.

## Data Availability

Please contact the author for data requests.

## Abbreviations

HNC: Head and neck cancers
VHNSS 2.0: The Vanderbilt Head and Neck Symptom Survey version 2.0
RT: Radiotherapy
QOL: Quality of life
HRQOL: Health-related quality of life
UW-QOL: University of Washington Quality of Life Questionnaire
EORTC QLQ-H&N35: European Organization for Research and Treatment of Cancer Quality of Life Questionnaire-H&N35
FACT-HN: Functional Assessment of Cancer Therapy Head and Neck Scale
CVI: Content validity index
AJCC: American Joint Committee on Cancer
IMRT: Intensity-modulated radiotherapy
RIOM: Radiation-induced oral mucositis
NPC: Nasopharyngeal carcinoma
